# Self-Monitoring by Traffic Light Color Coding Versus Usual Care on Outcomes of Patients With Heart Failure Reduced Ejection Fraction: Protocol for a Randomized Controlled Trial

**DOI:** 10.2196/resprot.9209

**Published:** 2018-11-14

**Authors:** Mahin Nomali, Ramin Mohammadrezaei, Abbas Ali Keshtkar, Gholamreza Roshandel, Shahrzad Ghiyasvandian, Kian Alipasandi, Masoumeh Zakerimoghadam

**Affiliations:** 1 Endocrinology and Metabolism Research Center Endocrinology and Metabolism Clinical Sciences Institute Tehran University of Medical Sciences Tehran Islamic Republic Of Iran; 2 Heart Failure Clinic Tehran Heart Center Tehran University of Medical Sciences Tehran Islamic Republic Of Iran; 3 Department of Health Science Education Development School of Public Health Tehran University of Medical Sciences Tehran Islamic Republic Of Iran; 4 Golestan Research Center of Gastroenterology and Hepatology Golestan University of Medical Sciences Gorgan Islamic Republic Of Iran; 5 Department of Medical Surgical Nursing School of Nursing and Midwifery Tehran University of Medical Sciences Tehran Islamic Republic Of Iran; 6 Department of Cardiology Tehran Heart Center Tehran University of Medical Sciences Tehran Islamic Republic Of Iran; 7 Department of Critical Care Nursing School of Nursing and Midwifery Tehran University of Medical Sciences Tehran Islamic Republic Of Iran

**Keywords:** heart failure, outcome, self-monitoring, clinical trial

## Abstract

**Background:**

Patients with heart failure (HF) reduced ejection fraction (HFrEF) have symptoms that are more severe and experience a higher rate of hospitalization compared with HF preserved ejection fraction (HFpEF) patients. However, symptom recognition cannot be made by patients based on current approaches. This problem is a barrier to effective self-care that needs to be improved by new self-monitoring instruments and strategies.

**Objective:**

This study describes a protocol for the self-monitoring daily diaries of weight and shortness of breath (SOB) based on the traffic light system (TLS). The primary objective is to compare the self-care between the intervention and control group. Comparison of HF knowledge, HF quality of life (HFQOL), and all-cause hospitalization between the 2 groups are the secondary objectives.

**Methods:**

A single-blind randomized controlled trial is being conducted at the HF clinic at Tehran Heart Center (Tehran, Iran). Sixty-eight adult patients of both genders will be enrolled during admission to HF clinic. Eligible subjects will be assigned to either the intervention or control group by a block balanced randomization method. Baseline surveys will be conducted before random allocation. Participants in the intervention group will receive an integrated package consisting of (1) HF self-care education by an Australian Heart Foundation booklet on HF, (2) regular home self-monitoring of weight and SOB, and (3) scheduled call follow-ups for 3 months. Patients in the control group will receive no intervention and they only complete monthly surveys.

**Results:**

This study is ongoing and is expected to be completed by the end of 2018.

**Conclusions:**

This is the first trial with new self-monitoring instruments in Iran as a low and middle-income country. If the findings show a positive effect, the package will be applied in different regions with the same health care status.

**Trial Registration:**

Iranian Registry of Clinical Trials IRCT2017021032476N1; https://en.irct.ir/trial/25296?revision=25296 (Archived by WebCite at http://www.webcitation.org/73DLICQL8)

**International Registered Report Identifier (IRRID):**

PRR1-10.2196/9209

## Introduction

Heart failure (HF) is a complex clinical syndrome that results from any structural and functional impairment of ventricular filling or ejection of blood. Based on left ventricle dysfunction and the left ventricle ejection fraction (LVEF), HF can be categorized as preserved ejection fraction (with LVEF≥50%, known as HFpEF or diastolic HF) and reduced ejection fraction (with LVEF ≤40%, known as HFrEF or systolic HF) [1].

As a global burden, HF affects more than 37.7 million individuals worldwide [2] and is a common condition with 1%-2% total health resources allocated in high-income countries. In low- and middle-income countries (LMICs) like Iran, HF may displace infectious diseases as the major public health issue [3]. In Iran and other Asian LMICs, HF is described as a pandemic [4], the prevalence greatly increased with age and range between 0.5% to 9.0% among different cities, regardless of time distribution [5], thus supporting the need for better HF surveillance and management strategies [3]. Patients with HF can experience various clinical outcomes including mortality, morbidity, and other patient-reported outcomes [6]. Although mortality and morbidity rates are similar for HFpEF and HFrEF, the hospital admission rates in patients with HFrEF are much higher than HFpEF [7].

HF is a leading cause of hospitalizations [8], mortality [8,9], rising health care costs (over US $39 billion annually in the United States and US $53.1 billion by 2030) [2,8-10], suboptimal self-care behavior [11], and reduced quality of life (QOL) [12]. According to a recent European Society of Cardiology Heart Failure Long-Term Registry prospective observational study, the rate of all-cause mortality and hospitalization at one-year follow-up was 8.1% and 28.2%, respectively [13]. Compared to Western countries, patients with HF in Asia and Iran are younger, have more severe signs and symptoms, and receive lower levels of treatment compared to the European Society of Cardiology and American College of Cardiology Foundation/American Heart Association Guidelines [14]. Moreover, Callender et al [3] reported that Iranian patients with HF experience significantly lower rates of admission (0.3%).

Poor self-care and low QOL among patients with HF are major global health issues [11] that are also affecting Iran [15-17]. Adequate self-care is associated with an improved health status [18], fewer symptoms, higher QOL [19], and reduced hospitalizations [18]. A lack of knowledge of symptom recognition and psychological problems have been identified as 2 key barriers [20]. Therefore, effective educational methods are essential for improved self-care [11,20]. Previous studies have shown that self-care programs for patients with HF are most effective when specialized education is combined with symptom monitoring and response management [21,22]. Practical and user-friendly self-monitoring tools can help patients recognize, monitor, report, and manage the symptoms appropriately. Without these tools, HF decompensation may not be detected in the early stages and could lead to preventable hospitalizations [23].

Patients with HF experience weight gain and other serious decompensation symptoms including shortness of breath (SOB) due to pulmonary edema, fatigue, and leg swelling [24-26]. Weight gain resulting from fluid retention is a marker of HF decompensation, and therefore, frequent weight monitoring can identify high-risk periods in the early stages to prevent hospitalization [24]. However, low compliance with weighing has been reported among patients with HF [27].

Diaries in a textual/tabular format are acceptable and creative self-monitoring tools because they reinforce patient education and facilitate their disease management [28]. Improved HF clinical and hospital outcomes have been reported by weight and symptom self-monitoring diary users [29]. However, despite the lower rate of HF-related hospitalizations [21,30], reduced mortality rate [21], and improved self-care [22], the previously reported studies used tabular format diaries and were conducted in developed countries with a higher quality of health care. The results may not apply to developing countries with significantly different health care systems and levels of health care quality.

Graph format diaries have been reported to attract and hold a person’s attention for more extended periods and are the preferred format due to improved patient comprehension and information extraction [31]. Recently, a traffic light system (TLS) of diaries in graphical format have been developed [32]. The system consists of 3 different color codes: (1) red for medical alert, (2) yellow for a situation that is worsening and requires the patient to take quick-relief medications, and (3) green to show that the patient is doing well and adequately adhering to the treatment plan [33]. The TLS has been shown to be more effective in transforming information on risk assessment and disease management [32].

Wakefield et al [23] employed a pretest-posttest longitudinal design study to assess weight, and SOB self-monitoring by graphical instrumentation (other than TLS) in patients with HF. The paper revealed that self-monitoring HF patients could not complete the appropriate actions for each zone. Consequently, new strategies and instruments to actively engage patients in symptom recognition and monitoring require further research [23].

This study will combine graphical and tabular formats with TLS color coding to design weight and SOB self-monitoring diaries. Each color zone has been designed for patients to perform specified actions following symptom monitoring. The efficacy of this intervention will be compared with the usual care for patients with HF. This is the first practical study with new user-friendly self-monitoring tools that will be conducted in Iran as an LMIC to compare the primary outcome of self-care behavior and the secondary outcomes of HF-knowledge, QOL, and all-cause hospitalization between intervention and control groups.

## Methods

### Study Design

This study is a parallel, single-blind, randomized controlled trial. The study protocol has been reviewed and approved (No. 9411449005) by the Institutional Review Board (IRB) at Tehran University of Medical Sciences (TUMS, Tehran, Iran). The study protocol is based on the Standard Protocol Items: Recommendations for Interventional Trials (SPIRIT) 2013 guideline [34].

### Study Setting

This study will be conducted at the Tehran Heart Center (THC) HF clinic affiliated with TUMS. THC is one of the best equipped diagnostic and therapeutic cardiology centers with full-time specialists and well-trained nursing staff in the region and is a referral center for cardiology in Iran with more than 1,300,000 outpatient visits, and 280,000 patient hospitalizations from 2001 to the end of 2017 [35].

### Eligibility Criteria

The research nurse (author MN) will screen the medical records of adult patients (≥18 years of age) of both genders for the following inclusion criteria in the HF clinic. The eligibility of each participant will be confirmed by the HF specialist (see Textbox 1).

### Intervention

In this study, the intervention is an integrated package consisting of 3 components: (1) HF self-care education, (2) regular home self-monitoring of weight and SOB, and (3) scheduled call follow-ups.

The first component of the package includes HF self-care education that will be delivered in the HF clinic of the THC according to the Australian Heart Foundation educational booklet entitled “living well with chronic HF” [38]. Individual education will be provided by the research nurse (MN) according to the participants understanding and literacy for a 40-minute face-to-face session between 8 am to 1 pm. A Farsi version of the booklet will be given to each participant. Also, during the educational session, the instructor and the participant will review the booklet content together, and the instructor will answer any questions. The booklet contains information on disease definition, diagnosis, symptoms, and ways to manage them, lifestyle issues, medicines, and other treatments. In order to obtain permission to translate the booklet to Farsi language for local use in Iran, one of the authors (MN) contacted the Australian Heart Foundation on April 5, 2016. After obtaining permission, it was translated to the Farsi language by an individual who was also proficient in the English language and knowledge of cardiovascular terminologies. Two nursing faculty members and 2 cardiology faculty members affiliated with TUMS approved the Farsi version of the booklet.

**Participant eligibility criteria.**

**Inclusion criteria**

- Definitive diagnosis of heart failure by an heart failure specialist documented on the clinical record
- Heart failure reduced ejection fraction where ejection fraction ≤40% [36,1] by any method (transthoracic, or transesophageal echocardiography, and angiography)
- New York Heart Association function class II-IV
- Access to a telephone, telegram mobile application, and ability to answer phone calls
- Ability to read and write in the Farsi language
- Having a functional weighing scale (digital or analog) at home to weigh daily [23]
- The absence of diagnosed hearing loss and sight defects
- A stable hemodynamic status
- Non-smoking, no drinking alcohol, and no addiction to drugs
- Living at home and not in a homeless shelter [37]

**Exclusion criteria**

- Diagnosed psychiatric and cognitive conditions during the study
- Sudden death, migration, or unavailability of patients during the follow-up phase
- Recording weight and shortness of breath in the daily diary for less than 60% (<54 days) during the study
- Undergoing cardiac or other kinds of surgery with a lengthy hospital stay during the study
- Awaiting or receiving a transplanted heart or a left ventricular assistive device

The second component of the package includes regular home self-monitoring of weight and SOB and recording them in the appropriate 3-color daily diaries for 3 months. Since the graphical display of values help patients to recognize their symptoms and contact their health care provider [23], we created a paper-based weight and SOB self-monitoring daily diary color-coded system that was analogous to TLS.

Based on previous studies, a graphical representation of health-related information is the preferred method for patients compared to numerical/tabular displays [39,40] for communicating medical risk information [41]. This approach results in better participant comprehension. The patient pays attention and extracts more information with less effort [31]. Thus, both research diaries have been designed in 3 colors and categorize the participants’ condition into 3 color-coded zones: green (excellent), yellow (use caution), and red (warning). The green, yellow, and red color-coded zones have been defined based on a combination of American Heart Association self-check plan for HF management [42] and the HF self-monitoring toolkit [43]. The colored diary will display recorded data in both graphical and tabular format. It is expected that this will result in a better participant understanding of their condition.

In the HF clinic, participants will be instructed by the research nurse on the daily measurement of weight and SOB and recording them in the daily diary during a 20-minute individual session between 8 am and 1 pm. The weight diary is a 7-page colored diary with instruction on the first page followed by 6 blank pages to record daily weight for 15 continuous days on each page.

**Figure 1:**
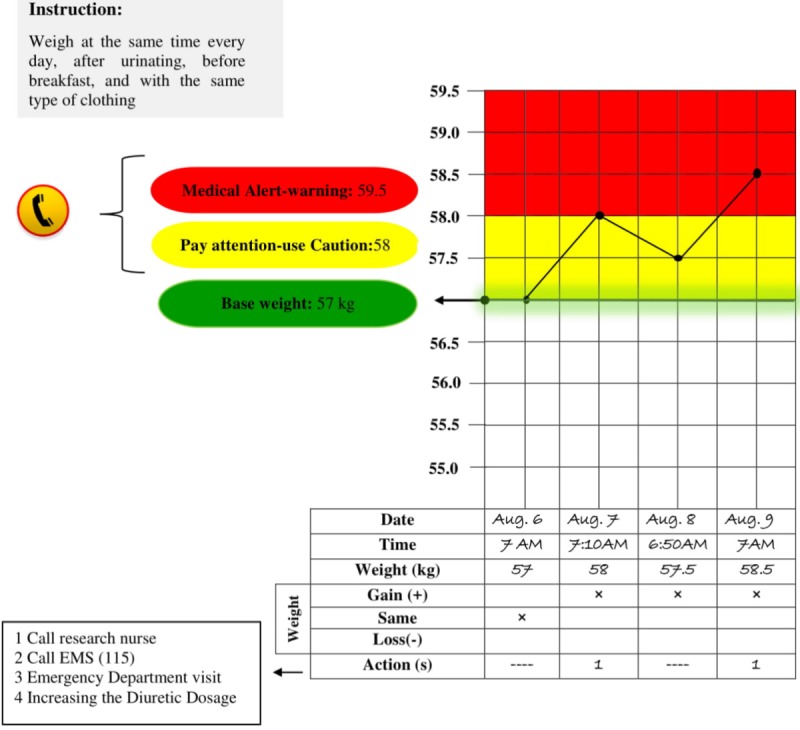
Colored weight daily diary. EMS: emergency medical services.

At the time of referral to the THC, the HF specialist will measure the weight of eligible participants by analog weight scale (RASA scale, model 230, Iran). Then, 2 upper control limits will be determined for participants by the research nurse to identify 1 and 2.5 kg greater than the baseline weight that is considered as yellow and red zones in the weight diary, respectively [23] (see Figure 1). During instruction, the participants will be asked to weigh themselves daily after going to the toilet, before breakfast, with the same type of clothing, at the same time [38] and record it regularly in the weight diary for 3 months. If participants miss a weight measurement for any reason, they will be asked to record the reason for the missed date. They also will be instructed to contact the research nurse in case of sudden weight gain of 1 kg in one day (yellow zone) and 2.5 kg in one week (red zone) [44].

The SOB diary is a 4-page colored diary that includes instructions on the first page and 3 blank pages to record the difficulty of breathing for 30 continuous days on each page. The participants will be instructed on how to use the SOB colored diary, and how to rate the difficulty of breathing for the entire day after taking evening medications and at the end of each day for 3 months [23]. They will also be instructed to record the reason for any missed measuring dates.

**Figure 2:**
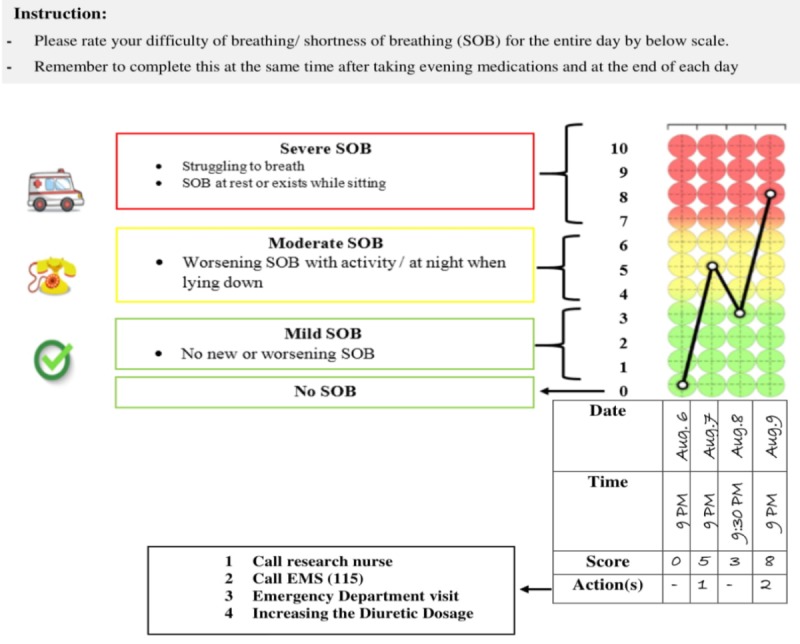
Colored shortness of breath daily diary. EMS: emergency medical services.

The SOB rating scale was designed based on 0 to 10 pain assessment visual analog scale. In this scale, scores are categorized as follows: none (0), mild (1-3), moderate (4-6), and severe (7-10) [45] that have been marked by green, yellow, and red colors, respectively. Green, yellow, and red color zones will be explained to participants as no new or worsening (none-mild SOB), worsening with activity or at night when lying down (moderate SOB), and struggling to breath or at rest or while sitting (severe SOB), respectively [42] (see Figure 2). Participants will be instructed to contact the research nurse if they reach the yellow color zone to increase the diuretic dosage and contact with the emergency medical services in the red color zone [38].

**Figure 3:**
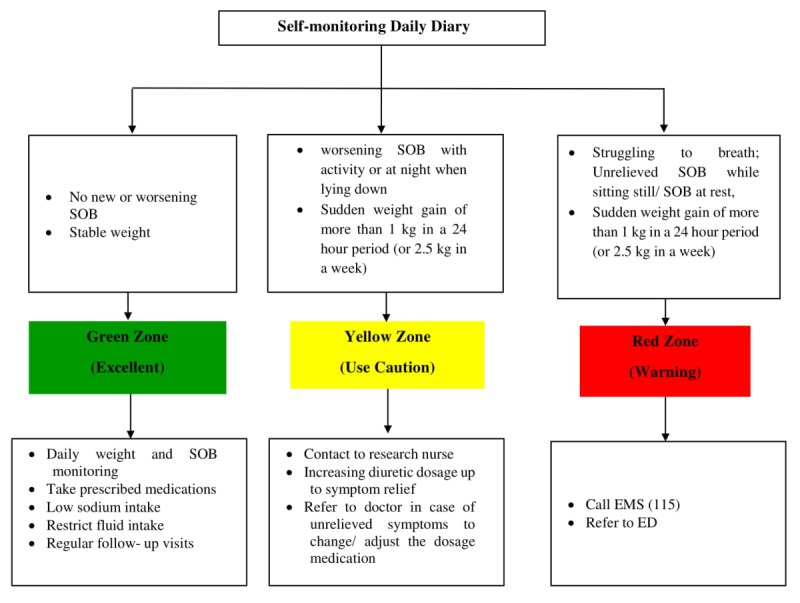
Flowchart of actions recommended by research nurse based on color zones of each subject's self-monitoring daily diary during call follow-ups. ED: emergency department; EMS: emergency medical services; SOB: shortness of breath.

**Figure 4:**
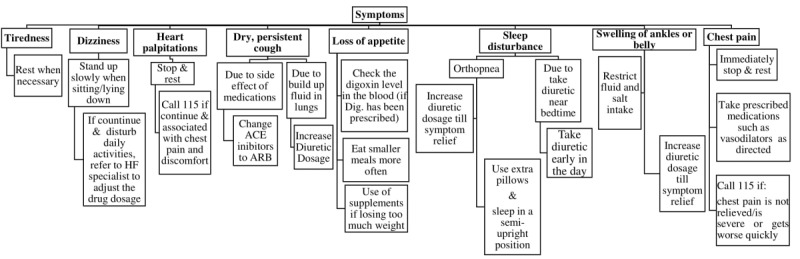
Flowchart of the research nurse recommendations for each symptom reported by subjects in the intervention group during the call follow-ups.

The third component of the package includes scheduled follow-up phone calls (days 1, 3, and 7 after referral to the HF clinic and every week after that) for 3 months [21]. The research nurse will give participants a list of call dates and will call each subject in the evenings between 6 pm and 9 pm. Each follow-up phone call will last 5-15 minutes [21] and is designed to review the content of the instructional session, encourage participants to continue weight and SOB self-monitoring, and record them in the daily diaries. Also, the research nurse will ask the participants about the color zone during the previous week and will recommend the appropriate action (see Figure 3). Also, we predict that during the call follow-ups, participants in the intervention group will report another symptom that may be due to a side effect of the medications or the disease. The research nurse will be able to provide the appropriate recommendation for each symptom reported in the intervention group during these (see Figure 4).

At the end of the one-hour instructional session in the HF clinic, a plastic folder containing the instructional booklet, weight, and SOB colored diaries, pen, pencil, eraser, and a pencil sharpener will be delivered to each subject in the intervention group. They will be asked to keep diaries clean and deliver them to the research nurse at the end of the study.

### Control

After randomization and allocating participants to 2 groups, those in the control group will receive the usual care. In HF clinic at THC, usual care for patients with HF is limited to a brief instruction by HF specialists on limiting water and salt intake, taking medications as prescribed, physical activity, weight monitoring and increasing the diuretic dosage in the condition of peripheral edema, weight gain, and SOB. They will receive 1 monthly phone call for 3 months. Out of respect for the ethical principles of human research, HF self-care instruction will be provided to the participants in the control group, and they will receive an educational booklet at the end of the study.

### Study Measures

#### Primary Outcome

##### Self-Care Behavior

Effective self-care improvement is a priority of all clinicians caring for patients with HF [46]. In a developing country like Iran, there is a growing need to improve patient’s self-care [15]. However, there is a low rate of reported admissions for HF in Iran [3]. Thus, in contrast to previous studies where hospitalization was considered the primary outcome [21,22,30] and self-care as the secondary outcome [21,22], self-care has been identified as the primary outcome in this trial.

Self-care behavior will be measured by the Self-Care of Heart Failure Index (SCHFI) version 6.2 [47] by self-reporting at baseline and monthly intervals for 3 months. SCHFI was translated into Persian and validated for Persian speaking people by Siabani et al in 2014 [48]. It consists of 3 separate scales and 22 questions: 10 for self-care maintenance, 6 for self-care management, and 6 for self-care confidence [49]. Cronbach alpha was determined for self-care maintenance (.56), management (.64), and confidence scales (.79) [48]. Scale scores can range between 0 to 100, and a score ≥70 will be considered as adequate self-care [49]. We will ask participants to complete SCHFI by themselves at baseline and then monthly for 3 months.

#### Secondary Outcomes

##### Heart Failure Knowledge

In order to assess the effect of education and counseling on knowledge of participants with HF, the Dutch Heart Failure Knowledge Scale (DHFKS), with Cronbach alpha=.62, will be used. This scale consists of a 15 multiple-choice self- administered questionnaire concerning: general knowledge on HF (4 questions), knowledge on HF treatment (6 questions on diet, fluid restriction, and activity), and symptoms and their recognition (5 questions). The participants will be asked to choose the correct option from the three choices. The minimum score for this scale can range from 0 (no knowledge) to a maximum score of 15 (excellent knowledge) [50].

While there is no Iranian version of this scale, it was translated into the Persian language by forward-backward translation methods after obtaining permission from the scale developer [50]. The face and content validity were evaluated by an expert’s opinion and discussion with patients with HF. The reliability was assessed through a pilot study among 30 patients with HF (Cronbach alpha=.62). This coefficient alpha was identical to the original one, and the internal consistency could not be improved by deleting any of the 15 items [50]. Thus, all items have been retained. Participants will be asked to provide answers to the DHFKS by themselves at baseline and the end of the study.

##### Quality of Life

Heart failure quality of Life (HFQOL) will be assessed by the Iranian version of the Minnesota Living with Heart Failure Questionnaire (MLHFQ) with Cronbach alpha=.95 [51]. The psychometric properties of the questionnaire were evaluated by Middel et al in 2001 (Cronbach alpha≥.80 for the scales) [52]. The MLHFQ consists of 21 questions with a 6-point Likert scale ranging from 0 (without limitation) to 5 (maximum limitation). It consists of physical and emotional dimensions. The physical dimension is the sum of the question scores (questions 1 to 7, 12, 13) while the emotional dimension is based on questions 17 to 21. Questions 8 to 11 and 14 to 16 are related to lifestyle, financial situations, and side effects of medications. The total is the sum of the 21-question score, and a lower score reflects better quality of life [53]. Since this instrument assesses HFQOL in the previous month [54], we will ask participants to complete MLHFQ by themselves at baseline and then monthly for 3 months.

##### All-Cause Hospitalization

Data related to all-cause hospitalization during the previous month (ie, cause, date of admission, discharge, and length of stay) will be obtained from participants in both groups by an outcome assessor blinded to study group assignment during monthly call follow-ups. In the case of admission to other health care settings except for THC, data will be collected according to the participants’ self-reporting. If the participant is admitted to THC, data will be confirmed by the hospital information system following participant self-reporting.

Causes of hospital admissions will be classified according to 3 categories of cardiac admission for HF: SOB and edema relieved by diuretics, other cardiac causes (ie, chest pain, arrhythmias, or syncope), and non-cardiac causes [21]. All-cause hospital admissions will be obtained by trained research assistants who are blinded to the allocation.

### Data Collection

Eligible patients will be asked to read and sign a written informed consent form. The research nurse will then collect demographic and clinical characteristics by participant self-reporting and from a medical chart and then record these in data collection forms (see Table 1). Also, participants will be asked to complete baseline surveys (SCHFI, HF knowledge, HFQOL, and health literacy) by themselves in the HF clinic before randomization.

In order to assess participant health literacy, we will use the Heart Failure-Specific Health Literacy Scale with Cronbach alpha=.71. It consists of 12 items each with 4 possible scores (1 to 4): inapplicable (1) to strongly applicable (4) where a higher score indicates a higher level of health literacy except items 1 to 4 [55]. The Iranian version of this scale was prepared after getting the developer’s permission. Thus, it was first translated into Persian by a forward-backward translation method. Then, face and content validity were approved by an expert’s opinion (nursing faculty, nurse practitioners, and cardiologists) and discussion with patients with HF. A pilot study was conducted to assess the reliability with 30 HF patients and where a Cronbach alpha value of .77 was determined. Deletion of items could not improve the value similar to the original scale. Therefore, we decided to retain all items [55]. We will ask the participants to complete the health literacy scale as a baseline survey.

In order to measure the HF knowledge at the end of the study, monthly self-care behavior, and the HFQOL, the outcome assessor will be blinded to study group assignments. The assessor will send the DHFKS, SCHFI, and MLHFQ to the participants in both groups by telegram mobile application in the morning of each monthly call follow-up and ask them to send answers before 6 pm of the same day.

### Participant Timeline

**Figure 5:**
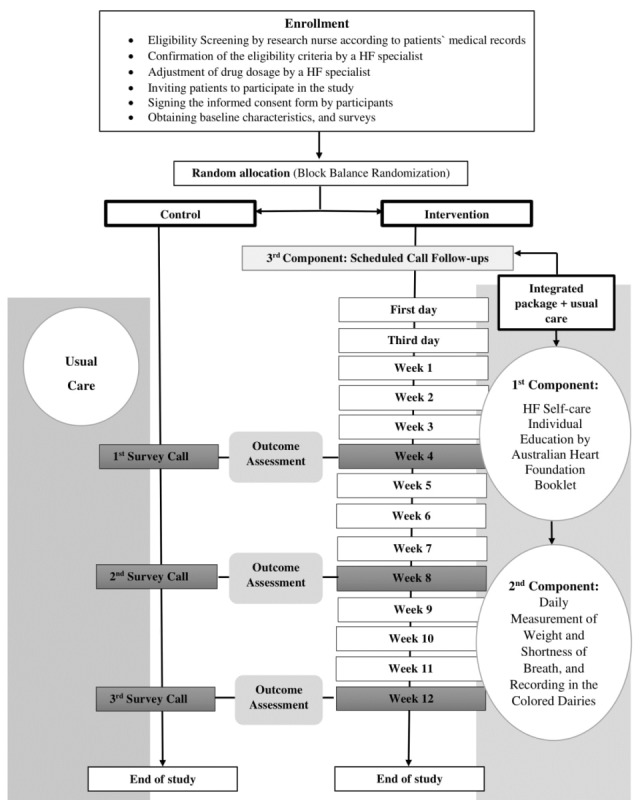
Schematic representation of the study design. HF: heart failure.

The SPIRIT 2013 template was used to schedule enrolment, interventions, and assessments (Table 1) [34]. A schematic representation of the study design is shown in Figure 5.

**Table 1:** Time schedule for enrolment, interventions, and assessments in this study.

Time point			Staff member	Study period						
				Enrollment: *-t*_*1*_^a^	Allocation: 0	Post-allocation				Close-out: *t_x_*^b^
						*t_1_* ^c^	*f*_1_	*f*_2_	*f*_3_^d^	
**Enrollment**										
	Eligibility screen		Research nurse and HF^e^ specialist	✓	—^f^	—	—	—	—	—
	Informed consent		Research nurse and HF specialist	✓	—	—	—	—	—	—
	Baseline characteristics and surveys		Research nurse	✓	—	—	—	—	—	—
Allocation			Research nurse	—	✓	—	—	—	—	—
**Intervention**										
	Integrated package plus usual care		Research nurse	—	—	✓	✓	✓	✓	—
**Control**										
	Usual care		—	—	—	✓	✓	✓	✓	—
**Assessments (demographic/clinical)**										
	Age (years)		Research nurse	✓	—	—	—	—	—	—
	Gender (male/female)		Research nurse	✓	—	—	—	—	—	—
	Ethnicity		Research nurse	✓	—	—	—	—	—	—
	Marital status (married/single)		Research nurse	✓	—	—	—	—	—	—
	Income		Research nurse	✓	—	—	—	—	—	—
	**Level of education**									
		Elementary	Research nurse	✓	—	—	—	—	—	—
		High school	Research nurse	✓	—	—	—	—	—	—
		Academic	Research nurse	✓	—	—	—	—	—	—
	HF-Specific Health Literacy Scale		Research nurse	✓	—	—	—	—	—	—
	Body mass index (kg/m^2^)		Research nurse	✓	—	—	—	—	—	—
	Job (yes/no)		Research nurse	✓	—	—	—	—	—	—
	Insurance (yes/no)		Research nurse	✓	—	—	—	—	—	—
	Time with HF		Research nurse	✓	—	—	—	—	—	—
	**HF etiology (yes/no)**									
		Ischemic heart disease	Research nurse	✓	—	—	—	—	—	—
		Cardiomyopathy	Research nurse	✓	—	—	—	—	—	—
		Hypertension	Research nurse	✓	—	—	—	—	—	—
		Heart valve disease	Research nurse	✓	—	—	—	—	—	—
	NYHA^g^ functional class (II–IV)		Research nurse	✓	—	—	—	—	—	—
	Previous hospitalization (yes/no)		Research nurse	✓	—	—	—	—	—	—
	Hospitalization last 3 months? (yes/no)		Research nurse	✓	—	—	—	—	—	—
	Charlson comorbidity index		Research nurse	✓	—	—	—	—	—	—
	Ejection fraction (%)		Research nurse	✓	—	—	—	—	—	—
	Systolic blood pressure (mm Hg)		Research nurse	✓	—	—	—	—	—	—
	Diastolic blood pressure (mm Hg)		Research nurse	✓	—	—	—	—	—	—
	Heart rate (bpm)		Research nurse	✓	—	—	—	—	—	—
	Daily weight measure (yes/no)		Research nurse	✓	—	—	—	—	—	—
	**Medications (yes/no)**									
		Angiotensin-converting enzyme inhibitor	Research nurse	✓	—	—	—	—	—	—
		Angiotensin receptor blocker	Research nurse	✓	—	—	—	—	—	—
		Diuretics	Research nurse	✓	—	—	—	—	—	—
		Aspirin	Research nurse	✓	—	—	—	—	—	—
		Statins	Research nurse	✓	—	—	—	—	—	—
		Vitamin k antagonists	Research nurse	✓	—	—	—	—	—	—
		Allopurinol	Research nurse	✓	—	—	—	—	—	—
		Beta blocker	Research nurse	✓	—	—	—	—	—	—
		Digitals	Research nurse	✓	—	—	—	—	—	—
	Implantable cardiac devices (yes/no)		Research nurse	✓	—	—	—	—	—	—
	Prior cardiac surgery? (yes/no)		Research nurse	✓	—	—	—	—	—	—
	Arrhythmia in electrocardiography? (yes/no)		Research nurse	✓	—	—	—	—	—	—
	**Laboratory parameters (serum)**									
		Sodium	Research nurse	✓	—	—	—	—	—	—
		Potassium	Research nurse	✓	—	—	—	—	—	—
		Urea	Research nurse	✓	—	—	—	—	—	—
		Creatinine	Research nurse	✓	—	—	—	—	—	—
		Uric acid	Research nurse	✓	—	—	—	—	—	—
		Hemoglobin	Research nurse	✓	—	—	—	—	—	—
		Hematocrit	Research nurse	✓	—	—	—	—	—	—
		Fasting blood glucose levels	Research nurse	✓	—	—	—	—	—	—
**Baseline surveys**										
	Self-Care of HF Index		Research nurse	✓	—	—	—	—	—	—
	Minnesota Living with HF Questionnaire		Research nurse	✓	—	—	—	—	—	—
	Dutch HF Knowledge Scale		Research nurse	✓	—	—	—	—	—	—
Primary outcome: Self-Care of HF Index			Research assistant	—	—	—	—	✓	✓	✓
**Secondary outcomes**										
	HF knowledge		Research assistant	—	—	—	—	—	—	✓
	HF quality of life		Research assistant	—	—	—	—	✓	✓	✓
	All-cause hospitalization (yes/no)		Research assistant	—	—	—	—	✓	✓	✓

^a^-*t*_1_: Time at enrollment.

^b^*t*_x_: Time at the end of study.

^c^*t*_1_: Time at allocation.

^d^*f*_1_: Time at first month follow-up; *f*_2_: Time at second month follow-up; *f*_3_: Time at third month follow-up.

^e^HF: heart failure.

^f^Not applicable.

^g^NYHA: New York Heart Association.

### Sample Size

In this study, the sample size was calculated based on the minimal clinically important difference (MCID) approach for self-care as measured by SCHFI for the primary outcome. No specific MCID for SCHFI was available to the best of our knowledge and based on our search of the PubMed database.

The suggested MCID for assessing dyspnea with the visual analog scale (0-100 score index) was the range 10-20 [56]. Thus, improving self-care for ≥10 scores from the baseline at the end of the study has been considered as MCID for the present study in order to reach a large sample size. According to the Jurgens et al [22] study in 2013, a mean of 70.8 for self-care in the control group at the end of study (90-days) and a fixed standard deviation of 27.2 (the largest SD) have been considered. In order to calculate sample size by G*Power free software (version 3.1.9.2), the input parameters are as following: effect size (Cohen *f*) of 0.168, the alpha error probability of .05, a statistical power of 80% to detect MCID≥10 scores between the 2 groups at the end of the study, 3 times measurement of outcome with monthly intervals, and a correlation coefficient of .5 among repeated measurements. Thus, by considering the overall attrition rates of 10%, the sample size was set at 68 participants (34 participants in each group).

### Participant Recruitment

During the run-in phase of the study, the research nurse at the HF clinic will be present Sunday through Wednesday of each week from 8 am to 1 pm. The nurse will screen the patients for the eligibility criteria at the time of admission. Participant screening will continue until the target population is achieved. Simultaneously, an HF specialist will confirm the subject`s eligibility and adjust the drug dosages of the eligible participants. Also, the specialist will provide each patient with additional details about the study. This is our main strategy for achieving adequate participant enrollment.

### Allocation: Method and Concealment Mechanism

The block balanced randomization method will be used for allocation. Before initiation of the run- in phase of the study, the statistician (AAK) will generate the randomization plan by website Randomization.com [57]. Therefore, twenty blocks of 4 for both the intervention group (group A) and the control group (group B) will be prepared. The statistician will fold the paper containing 4-size blocks 2 times, put them in the standard envelopes and write the serial numbers on them. All envelopes will be kept at the recruitment center. Thus, for every eligible patient, the research nurse randomly selects one of the envelopes after shuffling and assign participants into intervention and control groups.

### Blinding

Due to the nature of the intervention, it is impossible to blind the participants assigned to the study groups to the data collector. However, the outcome assessor will be blinded and instructed not to ask the participants whether or not they received the intervention.

### Data Management

Data will be collected in paper format. A file for each subject will be stored in numerical order in a secure place and manner. Files will be maintained at the recruitment center for 3 years after completion of the study. The statistical expert will prepare the data generated by the IBM/SPSS statistical software (version 16). This expert will train the data clerk to enter the data by specific codes and labels. Also, after finishing the data entry, all data will be double checked. All forms related to the study will be kept in a locked cabinet with restricted access in the recruitment center. All reports will be prepared in a manner in which no individual participants can be identified.

### Data Monitoring

Three researchers from TUMS not involved in the study and chosen by the university will monitor data and supervise the conduct of the study.

### Statistical Methods

Statisticians will prepare the data sheets to collected data. Data will be entered into the SPSS software (version 16). The Kolmogorov-Smirnov test will be used to normality. A transformation approach will be used to handle non-normal distribution. For the between-group comparison of continuous and categorical baseline characteristics, independent *t* test and chi-square test will be used. For categorical variables between the 2 groups, the two-tailed Fisher exact test will be used instead of the chi-square test in the case of too small expected cell frequencies. A *P* value <.05 will be considered statistically significant.

#### Primary Analysis

Baseline scores for each scale of SCHFI including self-care maintenance, management, and confidence will be compared in both groups by an independent *t* test. Due to the measurement of self-care behavior at baseline and at monthly time intervals, we will calculate the mean of monthly self-care scores in order to compare the mean of self-care behavior after the intervention in both groups. Also, because of the frequent measurement of self-care behavior, we will also use *P* value correction based on the Bonferroni method [58]. A linear logistic regression procedure will be applied to determine the factors affecting self-care behavior. The odds ratio will be reported with 95% CI.

#### Secondary Analysis

Independent *t* tests between 2 groups will compare the mean of the HF knowledge score before and at the end of the study. The HFQOL at baseline and at monthly time intervals will be compared by the *P* value correction based on the multiple comparisons.

In order to describe the surveillance, the median of admissions to health care settings will be used. Also, the Kaplan-Meier survival analysis will be applied for a descriptive comparison of the surveillance and, a log-rank test will be used to detect the significance difference between the 2 groups. A hazard ratio will be reported with 95% CI.

### Ethics Approval and Consent to Participate

The study protocol, data collection forms, educational booklet, and the template of informed consent form was reviewed and approved by the Research Ethics Committee (REC) at TUMS on February 5, 2017 (No. IR.TUMS.FNM.REC.1395.1653). Researchers are committed to updating the agreement with the IRB and REC at TUMS for any administrative and methodological changes of the study protocol which may affect the study.

In order to obtain the informed consent, the research nurse and HF specialist will introduce the trial to each eligible participant. Every eligible participant will receive an information sheet and a written informed consent. The research nurse will then obtain written informed consent from all participants willing to participate in the trial.

The participant informed consent form will be prepared according to the Declaration of Helsinki. It gives the subject information on what the research is about and what happens following participation in the study. It contains information on “purpose of the trial, potential benefits and risks; subject`s right to refuse participation or to withdraw consent at any time; institutional affiliation and potential competing interests of the researcher; and sources of trial funding” [59].

One critical issue that the researchers will be experiencing is posttrial care. Due to the nature of the intervention, this trial will not have any harm for participants that need to be treated during or after the trial. However, the HF specialist is the responsible physician and will assess and manage participants in the event of worsening of the symptoms.

### Availability of Data and Material

All study-related forms will be stored securely in the recruitment center by code number. Informed consent containing names and other personal identifiers will be stored separately from the forms identified by code number.

Intellectual property of the data belongs to the TUMS research and technology deputy. Thus, the final trial dataset/analysis will be available from the corresponding author upon request and by permission of the TUMS research and technology deputy.

## Results

This paper is the first version of the protocol that has been submitted. At the time of manuscript submission, the trial is actively enrolling the participants, and the recruitment started on June 11, 2017. The study is expected to be completed in November 2017. Data will be analyzed and published by the end of 2018.

## Discussion

The major concerns among patients with HF are the lack of information on the medications, worrying about both the quantity and combination of prescribed drugs, having trouble differentiating between the side effects of medications and symptoms of HF, and the lack of knowledge required to interpret symptoms or treat worsening symptoms [60]. The most critical participant issues are poor accessibility to a health care provider, receiving poor follow up, and having poor medication adherence [61].

Due to the nature of the intervention and the application of an integrated package including self-care educational booklet, graphical self-monitoring diaries based on TLS, and scheduled call follow-ups, we expect that this trial will address the patients’ concerns and issues.

We anticipate that the self-care educational booklet will improve the patient`s knowledge of HF. Graphical colored self- monitoring weight and SOB diaries will improve the patients’ ability to recognize their HF symptoms and help them determine the appropriate action whenever symptoms worsen. Also, scheduled call follow-ups will allow the patients to ask questions related to the disease, treatment options, and adverse effects. This will help them with referral to the proper health care setting in the early stages of the disease decompensation.

Iran is an LMIC and its health care system provides for only a brief verbal self-care instruction without any follow-up calls to patients with HF. Thus, at the end of this trial, patients in the intervention group and their caregivers may feel they are being deprived of standard care and a lack of support.

Thus, if the research findings provide positive effects, this practical intervention will be implemented for patients with HF in Iran and other countries similar to Iran. Also, the results of the research project plus the integrated package will be delivered to the noncommunicable diseases center at the Iran Ministry of Health, Education, and Treatment for consideration in the usual care of patients with HF.

## Abbreviations

DHFKS: Dutch Heart Failure Knowledge Scale
HF: heart failure
HFpEF: heart failure preserved ejection fraction
HFQOL: heart failure quality of life
HFrEF: heart failure reduced ejection fraction
IRB: institutional review board
LMIC: low- and middle-income country
LVEF: left ventricle ejection fraction
MCID: minimal clinically important difference
MLHFQ: Minnesota Living with Heart Failure Questionnaire
NYHA: New York Heart Association
QOL: quality of life
REC: research ethical committee
SCHFI: Self-Care Heart Failure Index
SOB: shortness of breath
SPIRIT: Standard Protocol Items: Recommendations for Interventional Trials
THC: Tehran Heart Center
TLS: traffic light system
TUMS: Tehran University of Medical Sciences
